# Supplementary material for: Analysis of PPARγ Signaling Activity in Psoriasis
Source: Int J Mol Sci. 2021 Aug 10;22(16):8603. doi: 10.3390/ijms22168603 (PMC8395241; doi:10.3390/ijms22168603)
Supplement: Supplementary file 1 [file ijms-22-08603-s001.zip › Supplemental materials_Analysis of PPARg signaling activity in psoriasis/Pathway models/Models images and html files/Differentiation of psoriatic T cells/48500.html]

SMURF2


# Protein SMURF2

|  |  |
| --- | --- |
| URN | urn:agi-llid:64750 |
| Total Entities | 0 |
| Connectivity | 653 |
| Name | SMURF2 |
| Description | SMAD specific E3 ubiquitin protein ligase 2 |
| Primary Cell Localization | Cytoplasm |

---

|  |  |
| --- | --- |
| Pathway | Osteoarthritis |
|  | TGF-beta Signaling in Skin Aging |
|  | Proteins Involved in Osteoarthritis |
|  | TGFB2 Signaling Impairement in Osteoarthritis |
|  | Proteins with Altered Expression in Osteoarthritis |
|  | Bone Loss in Osteoporosis |
|  | Breast Cancer |
|  | Ovarian Cancer |
|  | BMP7/ACVR2 Expression Targets |
|  | TGFB Signaling activation by Blocking of Tumor Suppressors |
|  | INHBA/ACVR2/ACVR1 Expression Targets |
|  | TGFBR/BMPR -> SMAD2/3 Signaling |
|  | ActivinR -> SMAD2/3 Signaling |
|  | TGFBR1/AP-1 Signaling |
|  | MSTN/ACVR2/ACVR1 Expression Targets |
|  | TGF-beta Signaling |
|  | TGFB2-TGFBR1 Expression Targets |
|  | TGFB3-TGFBR1 Expression Targets |
|  | TGFB1-TGFBR1 Expression Targets |
|  | TGFB1-TGFBR1/AP-1 Expression Targets |
|  | 1\_Differentiation of psoriatic T cells |
|  | 3\_TGFB signaling provokes endochondral ossification with osteophyte formation |
|  | Differentiation of psoriatic T cells |

---

|  |  |
| --- | --- |
| MedScan ID | 64750 |

---

|  |  |
| --- | --- |
| LocusLink ID | 64750 |
|  | 66313 |
|  | 303614 |

---

|  |  |
| --- | --- |
| Alias | SMURF2 |
|  | MGC138150 |
|  | HECT-type E3 ubiquitin transferase SMURF2 |
|  | DKFZp686F0270 |
|  | 2810411E22 |
|  | smad specific e3 ubiquitin protein ligase 2 |
|  | hSMURF2 |
|  | smad specific e3 ubiquitin protein ligase II |
|  | E3 ubiquitin ligase SMURF2 |
|  | 2810411E22Rik |
|  | SMAD ubiquitination regulatory factor 2 |
|  | RP23-170A23.3 |
|  | AI558114 |
|  | AI649275 |
|  | RGD1310067 |
|  | OTTMUSP00000003747 |
|  | E3 ubiquitin-protein ligase SMURF2 |
|  | SMAD-specific E3 ubiquitin-protein ligase 2 |

---

|  |  |
| --- | --- |
| EC Number | 2.3.2.26 |
|  | 6.3.2.- |

---

|  |  |
| --- | --- |
| GO ID | 0046332 |
|  | 0005160 |
|  | 0061630 |
|  | 0004842 |
|  | 0030509 |
|  | 0060071 |
|  | 0030514 |
|  | 0000122 |
|  | 0045892 |
|  | 0030512 |
|  | 0090263 |
|  | 0045732 |
|  | 1901165 |
|  | 0043161 |
|  | 0016579 |
|  | 0000209 |
|  | 0016567 |
|  | 0017015 |
|  | 0030579 |
|  | 0006511 |
|  | 0005737 |
|  | 0005829 |
|  | 0045121 |
|  | 0016607 |
|  | 0005654 |
|  | 0005634 |
|  | 0005886 |
|  | 0000151 |
|  | 0016740 |
|  | 0016020 |
|  | 0016874 |
|  | 0042787 |
|  | 0008766 |
|  | 0043773 |
|  | 0043774 |
|  | 0070739 |
|  | 0070735 |
|  | 0070737 |
|  | 0070736 |
|  | 0018169 |
|  | 0070740 |
|  | 0070738 |
|  | 0007179 |
|  | 0010467 |
|  | 0006367 |
|  | 0006351 |

---

|  |  |
| --- | --- |
| KEGG ID | hsa:64750 |
|  | mmu:66313 |

---

|  |  |
| --- | --- |
| Organism | Homo sapiens {Organism urn:agi-taxid:9606} |
|  | Mus musculus {Organism urn:agi-taxid:10090} |
|  | Rattus norvegicus {Organism urn:agi-taxid:10116} |
|  | Homo sapiens |
|  | Mus musculus |
|  | Rattus norvegicus |

---

|  |  |
| --- | --- |
| Mouse chromosome position | 11 |

---

|  |  |
| --- | --- |
| OMIM ID | 605532 |

---

|  |  |
| --- | --- |
| Rat chromosome position | 10q32.1 |

---

|  |  |
| --- | --- |
| Hugo ID | 16809 |
|  | HGNC:16809 |

---

|  |  |
| --- | --- |
| Human chromosome position | 17q23.3-q24.1 |
|  | 17q22-q23 |

---

|  |  |
| --- | --- |
| Swiss-Prot Accession | Q96DE7 |
|  | Q9HAU4 |
|  | Q9HAU4.1 |
|  | A2A5Z6 |
|  | Q9CSE3 |
|  | A2A5Z6.1 |
|  | Q52LL1 |
|  | Q9H260 |
|  | A2A5Z7 |
|  | Q5IRE6 |

---

|  |  |
| --- | --- |
| GenBank ID | NC\_000017 |
|  | NM\_022739 |
|  | NP\_073576 |
|  | XM\_005257585 |
|  | XP\_005257642 |
|  | AC009994 |
|  | AC132812 |
|  | CH471109 |
|  | EAW94193 |
|  | EAW94194 |
|  | EAW94195 |
|  | HF584194 |
|  | CCQ43691 |
|  | KF456306 |
|  | AF301463 |
|  | AAG25641 |
|  | AF310676 |
|  | AAG45422 |
|  | AK002019 |
|  | AY014180 |
|  | AAG50421 |
|  | BC009527 |
|  | AAH09527 |
|  | BC034789 |
|  | BC051283 |
|  | BC093876 |
|  | AAH93876 |
|  | BC111945 |
|  | AAI11946 |
|  | BP363931 |
|  | CB267500 |
|  | CN310238 |
|  | CN370887 |
|  | CR936721 |
|  | Q9HAU4 |
|  | NC\_000077 |
|  | XM\_006533936 |
|  | XP\_006533999 |
|  | XR\_001780039 |
|  | NM\_025481 |
|  | NP\_079757 |
|  | XM\_017314705 |
|  | XP\_017170194 |
|  | NM\_001362894 |
|  | AL593847 |
|  | CH466558 |
|  | EDL34316 |
|  | AK012060 |
|  | AK013082 |
|  | BAB28637 |
|  | AK020616 |
|  | AK036718 |
|  | AK161516 |
|  | BAE36438 |
|  | AY685230 |
|  | AAV87906 |
|  | BC023703 |
|  | BC051489 |
|  | AAH51489 |
|  | BC065796 |
|  | AAH65796 |
|  | BC099525 |
|  | BC138786 |
|  | AAI38787 |
|  | BC138788 |
|  | AAI38789 |
|  | BE986450 |
|  | BU058366 |
|  | BU614468 |
|  | A2A5Z6 |
|  | NC\_005109 |
|  | NM\_001107061 |
|  | NP\_001100531 |
|  | XM\_017597342 |
|  | XP\_017452831 |
|  | AC\_000078 |
|  | AABR07030590 |
|  | AABR07030591 |
|  | AABR07030592 |
|  | AABR07030593 |
|  | AAHX01066287 |
|  | AAHX01066288 |
|  | AAHX01066289 |
|  | AAHX01066290 |
|  | CH473948 |
|  | EDM06417 |
|  | XM\_011525128 |
|  | XP\_011523430 |
|  | NC\_018928 |
|  | AMYH02033792 |
|  | AMYH02033793 |
|  | XM\_006533938 |
|  | XP\_006534001 |
|  | XM\_011249176 |
|  | XP\_011247478 |
|  | AC\_000033 |
|  | AAHY01096058 |
|  | AAHY01096059 |
|  | XM\_008768365 |
|  | XP\_008766587 |
|  | AC\_000149 |
|  | ABBA01035289 |
|  | ABBA01035290 |
|  | ABBA01035291 |
|  | ABBA01035292 |
|  | ABBA01035293 |
|  | ABBA01035294 |
|  | XM\_006533935 |
|  | XP\_006533998 |
|  | XM\_006533937 |
|  | XP\_006534000 |

---

|  |  |
| --- | --- |
| Swiss-Prot ID | SMUF2\_HUMAN |
|  | SMUF2\_MOUSE |

---

|  |  |
| --- | --- |
| Cell Localization | Nucleus |
|  | Cytoplasm |
|  | Cell membrane |
|  | Membrane raft |

---

|  |  |
| --- | --- |
| Ensembl ID | ENSG00000108854 |
|  | ENSP00000262435.9 |
|  | ENST00000262435.14 |
|  | ENSMUSG00000018363 |
|  | ENSMUSP00000090177.2 |
|  | ENSMUST00000092517.8 |
|  | ENSMUSP00000099356.3 |
|  | ENSMUST00000103067.9 |
|  | ENSRNOG00000014623 |
|  | ENSRNOP00000019781.4 |
|  | ENSRNOT00000019781.6 |
|  | ENSP00000262435 |
|  | ENST00000262435 |
|  | ENSMUSP00000090177 |
|  | ENSMUST00000092517 |
|  | ENSMUSP00000099356 |
|  | ENSMUST00000103067 |
|  | ENSRNOP00000019781 |
|  | ENSRNOT00000019781 |

---

|  |  |
| --- | --- |
| MGI ID | MGI:1913563 |

---

|  |  |
| --- | --- |
| RGD ID | 1310067 |

---

|  |  |
| --- | --- |
| Unigene ID | Hs.515011 |
|  | Mm.340955 |
|  | Rn.92843 |

---

|  |  |
| --- | --- |
| Homologene ID | 41490 |

---
